# Diagnosis and Management of an Adenomatoid Uterine Tumor: Ultrasound, Magnetic Resonance Imaging, Surgical Appearance, and Pathology Correlation

**DOI:** 10.1089/biores.2018.0023

**Published:** 2018-10-26

**Authors:** Jennie Orlando, Cornelia deRiese, Eric Blackwell, Suzanne Graham, Jennifer Phy

**Affiliations:** ^1^ Department of Obstetrics and Gynecology and Texas Tech University Health Sciences Center, Lubbock, Texas.; ^2^ Department of Pathology, Texas Tech University Health Sciences Center, Lubbock, Texas.

**Keywords:** adenomatoid, gynecology, leiomyoma

## Abstract

Adenomatoid uterine tumors are rare, and their appearance on medical imaging modalities is not well established. We present a case of an adenomatoid uterine tumor reviewing a unique sonographic presentation, magnetic resonance imaging (MRI), gross surgical appearance of the tumor, and microscopic pathology images. A 29-year-old gravida 0 Caucasian woman presented with dysmenorrhea, menorrhagia, and desire to conceive. Transvaginal ultrasound revealed a 2.7 cm round, well-circumscribed posterior intramural uterine mass. The mass was hyperechoic centrally with a thin hypoechoic rim. Color Doppler imaging revealed a prominent vascular rim around the periphery of the mass as well as central vascularity not typical for a leiomyoma. MRI, with and without intravenous gadolinium, was obtained showing a 2.7 cm posterior fundal mildly enhancing uterine mass suggestive of leiomyoma. The mass had a heterogeneous signal pattern on T2-weighted images, and no fat component was noted within the mass. Repeat transvaginal ultrasound showed interval growth of the mass to 3.5 cm with a lipomatous appearance. Adenomatoid uterine tumors are rare and may be mistaken for uterine leiomyomata. Unique features include sonographic appearance of central hyperechogenicity with a hypoechoic rim and prominent peripheral and central vascularity in conjunction with MRI revealing a heterogeneous signal pattern on T2-weighted images without fat component. Gross surgical appearance reveals a nondiscrete capsule and secretion of mucoid material when the mass is exposed. We present a case of adenomatoid tumor providing sonographic, MRI, surgical, and pathological correlation. The patient subsequently conceived spontaneously and delivered at term by cesarean section. The patient underwent a preoperative evaluation with complete blood count, comprehensive metabolic panel, blood type with antibody screen, and pregnancy test. She underwent laparoscopic excision with robotic assistance for removal of the tumor. Grossly, the uterine mass had a very soft consistency atypical for a uterine leiomyoma making dissection more challenging. During dissection the mass diffusely secreted a mucoid material although the capsule was not disrupted. The lesion was excised intact and was removed from the peritoneal cavity in an endocatch bag without internal morcellation. Microscopic examination revealed an adenomatoid tumor.

## Introduction

Uterine tumors are commonly identified in gynecological patients and are often linked with symptoms of painful and irregular menses. The majority of these uterine tumors are leiomyomas that occur in 20–25% of women (William's, 2012, p. 247).^1^ However, other uterine tumors may be identified, including leiomyosarcomas, lipoleiomyomas, or adenomatoid tumors. Adenomatoid tumors are benign growths of the uterine serosa and myometrium originating from the mesothelium and forming gland-like structures.^2^ Adenomatoid tumors are often discovered incidentally in pathology specimens and may be mistaken clinically for typical fibroids.^16^ We present a case of an adenomatoid uterine tumor reviewing a unique sonographic presentation, magnetic resonance imaging (MRI), gross surgical appearance of the tumor, and microscopic pathology images.

## Methods

**FIG. 1.**
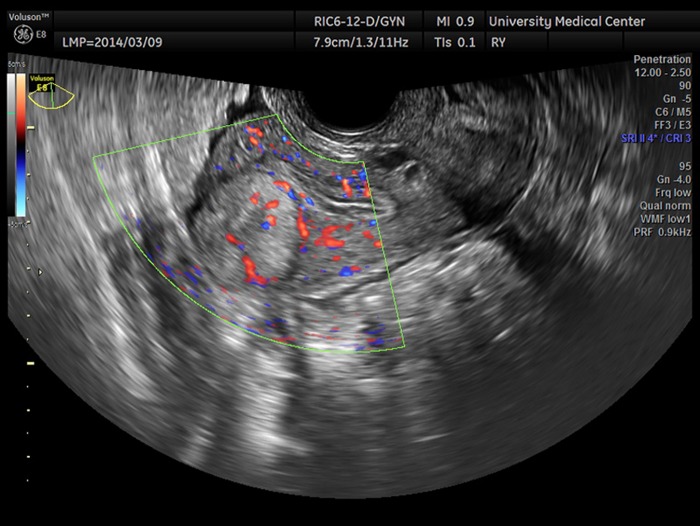
Transverse transvaginal ultrasound image of the upper aspect of the uterus obtained on March 17, 2014, showing a 2.74 cm mostly echogenic mass with a hypoechoic rim extending from the serosa to the endometrium.

**FIG. 2.**
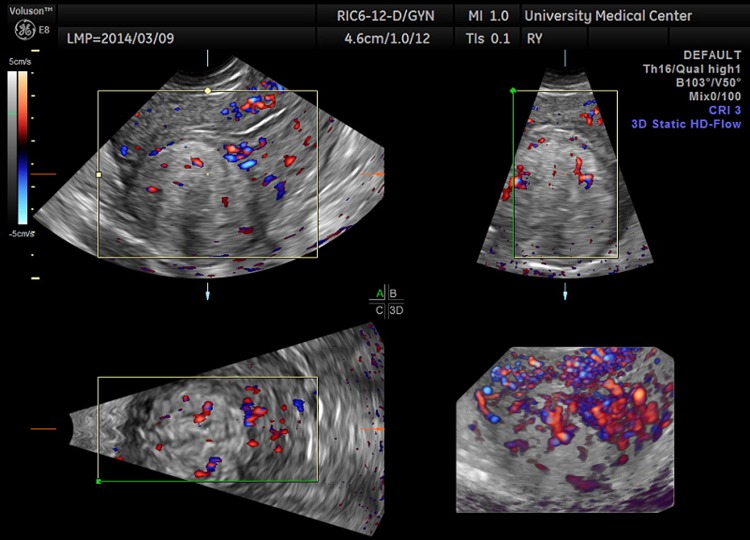
Orthogonal three-dimensional transvaginal ultrasound images of the uterus (with color Doppler overlay) showing vascularity both within and at the periphery of the mass.

**FIG. 3.**
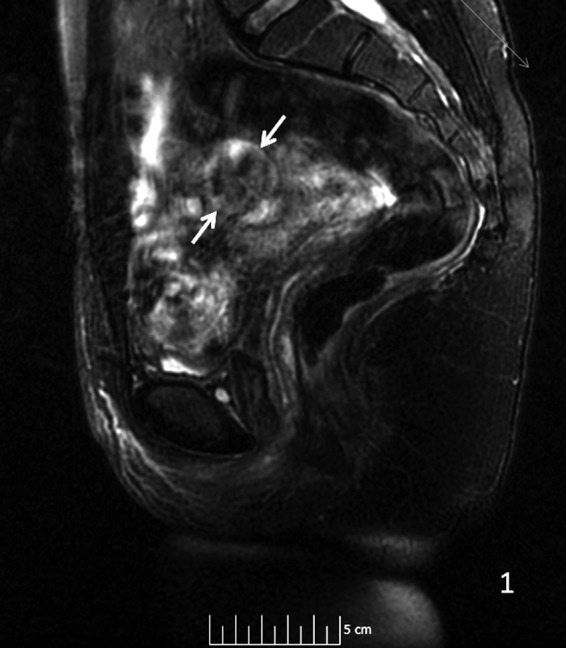
Sagittal T2 MRI shows arrows pointing to an in homogeneous pattern in the mass. MRI, magnetic resonance imaging.

**FIG. 4.**
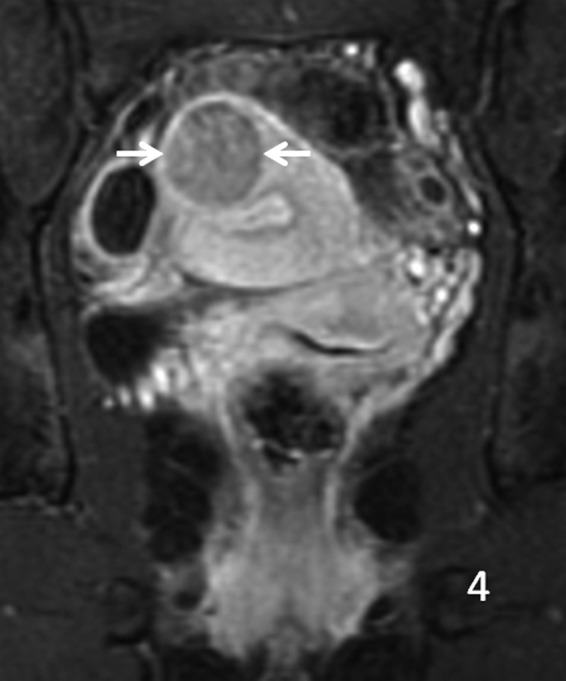
Coronal T1 MRI with contrast, arrows showing mild signal enhancement.

**FIG. 5.**
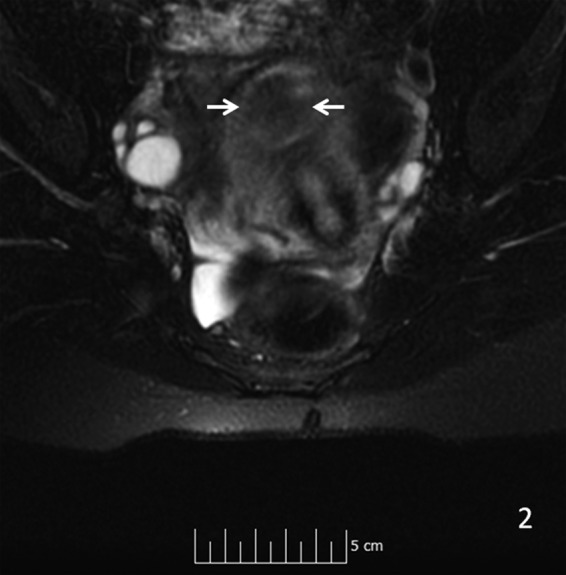
Axial T2 MRI shows arrows pointing to an intermediate signal strength in the mass.

**FIG. 6.**
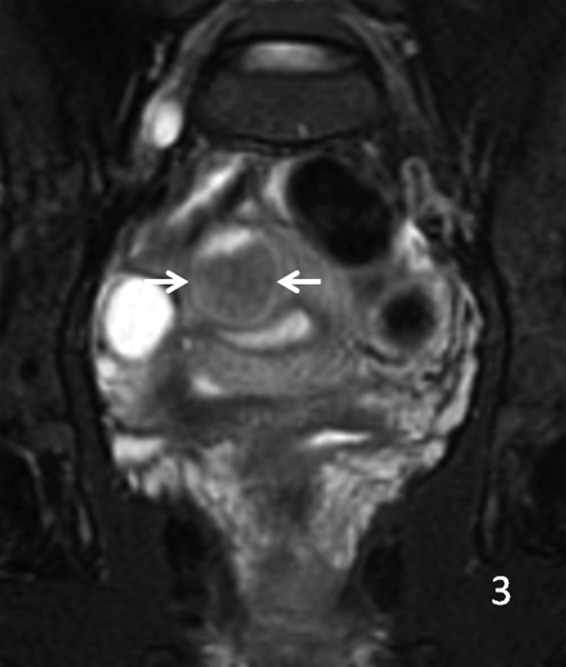
Coronal T2 fat saturation MRI image, with arrows showing signal strength in the mass.

A 29-year-old gravida 0 Caucasian woman presented with dysmenorrhea, menorrhagia, and desire for conception. The first transvaginal ultrasound on March 17, 2014 revealed a 2.8 × 2.7 × 2.6 cm echogenic spherical subserosal uterine mass in the right fundal area. The mass was hyperechoic centrally with a thin hypoechoic rim, which is typical for a lipomatous uterine tumor such as a lipoleiomyoma (Fig. 1). However, color Doppler imaging revealed a prominent vascular rim around the periphery of the mass and many vessels within the mass, not typical for a leiomyoma (Fig. 2). Multisequence MRI, with and without intravenous gadolinium, was obtained on April 14, 2014 showing a 2.7 × 2.7 cm posterior fundal mildly enhancing uterine mass suggestive of leiomyoma. The mass had heterogeneous signal pattern on T2-weighted images, and no fat component was noted within the mass (Figs. 3–6). Repeat transvaginal ultrasound 5 months later in September showed interval growth of the mass to 3.8 × 3.6 × 3.1 cm with continued evidence of a lipomatous appearance despite the absence of any fat component on the MRI scan.

## Results

**FIG. 7.**
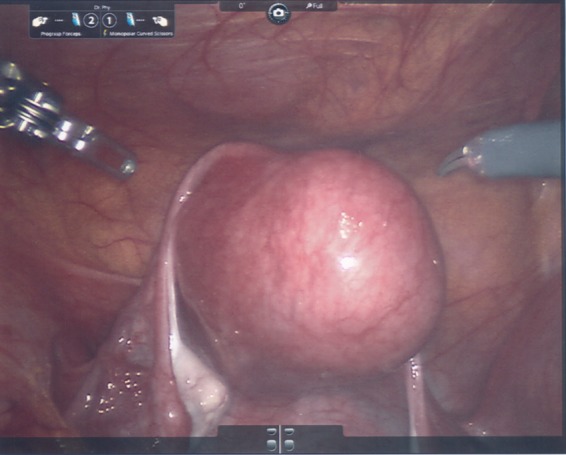
Laparoscopic image taken at time of surgery shows the uterus with the mass inside.

**FIG. 8.**
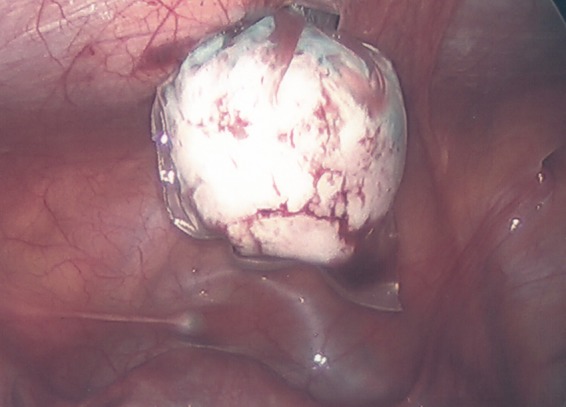
Laparoscopic image showing uterine mass removed and placed inside the endocatch bag for removal through trocar port site.

**FIG. 9.**
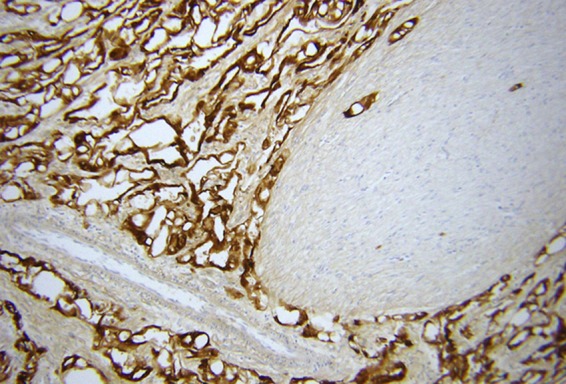
Stain for calretinin decorates the lining of the gland-like spaces, confirming the cells to be mesothelial in the absence of Ber-EP4 staining (immumoperoxidase stain, original magnification 125× ).^5^

**FIG. 10.**
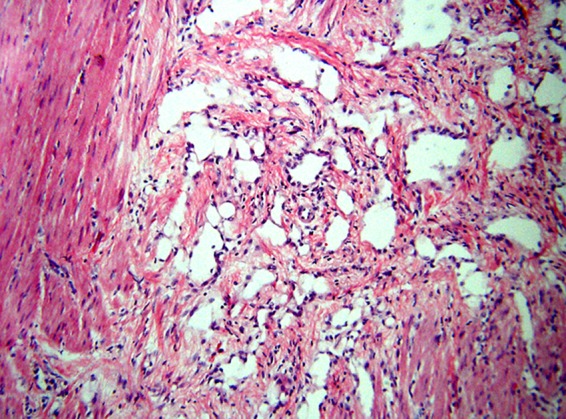
A sieve-like pattern of apparently cell-lined empty gland-like spaces surrounds fascicle of smooth muscle (hematoxylin and eosin, original magnification 125× ).

The mass was removed through laparoscopic excision with robotic assistance. Grossly, the uterine mass had a very soft consistency, atypical for a uterine leiomyoma making dissection more challenging (Figs. 7 and 8). The mass diffusely secreted a mucoid material during dissection but was excised intact and was removed from the peritoneal cavity in an endocatch bag without internal morcellation. Microscopic examination revealed and confirmed an adenomatoid tumor (Figs. 9 and 10). Gross inspection of the specimen revealed a 3.5 × 3.0 × 2.8 cm well-circumscribed trabeculated tan-white tissue mass with a mucoid cut surface. The surface was spongy in appearance and texture. Microscopic pathological evaluation revealed a fascicular proliferation of smooth muscle tissue punctuated by innumerable collapsed gland-like spaces lined by flattened cells with little appreciable cytoplasm, forming sieve-like areas in some regions. Original magnification of specimens was 25× ; however, with hematoxylin and eosin, original magnification started at 125× . An immunoperoxidase panel confirmed that the spaces of interest were lined by mesothelial cells, being positive for a marker for mesothelium and calretinin. Immunoperoxidase stain for calretinin started at 125× magnification, and was negative for a marker of epithelial/glandular cells, Ber-EP4, and negative for CD34, a marker used in this case for endothelial cells.

## Discussion

Adenomatoid tumors are found in the reproductive organs of both men and women. They are commonly incidental and diagnosed by histology postoperatively. The study of adenomatoid tumors began with Golden and Ash.^3^ “The primary unit of the tumor is epithelial in nature and it tends to form gland-like spaces,” which was suggested later on by Gordon-Taylor and Ommaney-Davis (Adenomatoid, p. 86–87).^4^ These tumors, although they appear disordered, have no evidence of invasion or metastasis. They are usually large and often slow growing. Evans, in the *American Journal of Pathology*, later went on to argue that the cells of an adenomatoid tumor are mesothelial in origin, which helps explain their locality in the reproductive tracts of men and women.

In a 2013 study of the incidence of uterine adenomatoid tumors, the authors reported a 1% incidence of adenomatoid tumors diagnosed on specimens after hysterectomy or tumor excision with uterine preservation. Their study showed a wide range of 29–67 years of age in women. It also showed that the tumors ranged from 0.5 to 7 cm. Preoperatively these tumors appeared as leiomyomas and it was not until the specimen was viewed by a pathologist that an adenomatoid tumor was diagnosed.

Adenomatoid uterine tumors are rare and are often mistaken for uterine leiomyomata. The rarity of adenomatoid tumors of the uterus has limited the opportunity for characterization of these masses by medical imaging modalities. Our case suggests that ultrasound findings typical of a lipomatous uterine mass, in conjunction with MRI findings showing absence of fat components, may be supportive of an adenomatoid uterine tumor.

Unique features include sonographic appearance of central hyperechogenicity with a hypoechoic rim and prominent peripheral and central vascularity on color Doppler images, whereas MRI reveals heterogeneous signal pattern on T2-weighted images without fat component. Gross surgical appearance reveals a nondiscrete capsule and secretion of mucoid material when the mass is exposed. We present a case of adenomatoid tumor providing sonographic, MRI, surgical, and pathological correlation. The patient subsequently conceived spontaneously and delivered at term.

## Abbreviation Used

MRI: magnetic resonance imaging

## References

[1] Hottman BL, Schorge JO, Schatter JI, *et al* Pelvic mass. In: Williams Gynecology, *2nd* *ed* Hoffman BL, Schorge JO, Schaffer JI, (eds.), McGraw-Hill Medical: New York; pp. 247; 2012

[2] Nakayama H, Teramoto H, Teramoto M True incidence of uterine adenomatoid tumors. Biomed Rep. 2013;1:352–354 24648947 10.3892/br.2013.72 PMC3917104

[3] Golden A, Ash JE Adenomatoid tumors of the genital tract. Am J Pathol. 1945;21:63–79 19970804 PMC1934086

[4] Gopinath A, Injody Susy J, Babu MA, *et al.* Adenomatoid tumor of the uterus: a rare leiomyoma mimicker. J Obstet Gynaecol India. 2011;61:86–87

[5] Said JW, Nash G, Lee M Immunoperoxidase localization of keratin proteins, carcinoembryonic antigen, and factor VIII in adenomatoid tumors: evidence for a mesothelial derivation. Hum Pathol. 1982;13:1106–1108 6184302 10.1016/s0046-8177(82)80247-5

